# Optical Modification of TMD Heterostructures

**DOI:** 10.1021/acs.nanolett.4c06512

**Published:** 2025-03-05

**Authors:** Suvi-Tuuli Varjamo, Christopher Edwards, Yaoqiang Zhou, Ruihuan Fang, Seyed Hossein Hosseini Shokouh, Zhipei Sun

**Affiliations:** QTF Centre of Excellence, Department of Electronics and Nanoengineering, Aalto University, Espoo 02150, Finland

**Keywords:** 2D materials, transition metal dichalcogenides, laser patterning, defect engineering, optical modification, anti-ambipolar transistor, ternary inverter

## Abstract

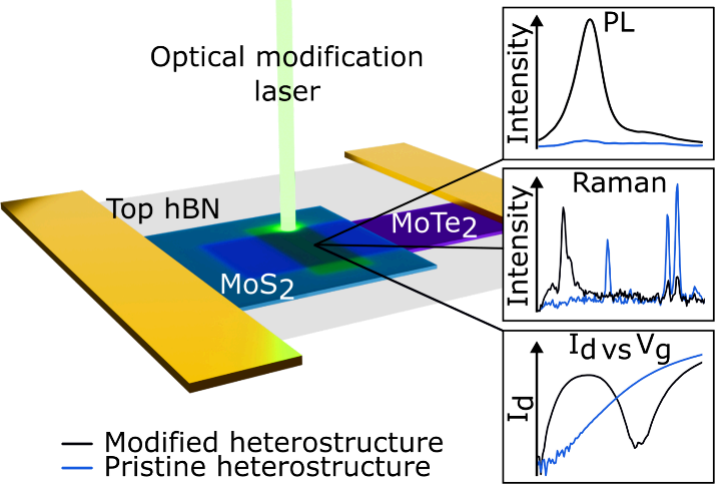

Optical modification is a fast, cost-effective, and scalable approach to tailoring the physical properties of two-dimensional (2D) materials for various applications. However, most previous efforts have focused on modifying individual 2D materials, which fails to utilize the method to its fullest potential. In this paper, heterostructures composed of hBN-capped molybdenum ditelluride (MoTe_2_) and molybdenum disulfide (MoS_2_) are optically modified with a continuous wave laser. The process simultaneously thins MoS_2_ and induces clustering of tellurium atoms from the ablated MoTe_2_. These structural changes result in significant enhancements of the physical properties, including a 43-fold increase in MoS_2_ photoluminescence and the transformation of the heterojunction into an anti-ambipolar transistor. These findings highlight a previously unutilized pathway to tune the heterostructure properties for applications in novel electronics and optoelectronics.

Two-dimensional (2D) materials have been studied extensively for the past 20 years ever since the exfoliation of monolayer graphene in 2004.^1^ One heavily investigated 2D material group is semiconducting transition metal dichalcogenides (TMDs), which consist of a transition metal (e.g., molybdenum (Mo), tungsten (W)) sandwiched between two chalcogen (e.g., sulfur (S), tellurium (Te)) atoms.^2^ TMDs have remarkable properties, including layer-dependent^2,3^ and gate-tunable^4^ band gap, high photoluminescence (PL) governed by excitons,^3^ and versatile electrical properties.^5^ Additionally, TMD heterostructures support interlayer excitons^6^ and form adaptable heterojunctions,^7−9^ making them optimal for applications in matrix and logic computing,^10^ memory devices,^11^ photodetectors,^12^ and spectrometers.^13,14^

Due to their atomic thickness and large surface-to-volume ratio, the properties of 2D materials can readily be modified. However, many conventional methods (e.g., plasma, electron beam irradiation, and thermal annealing)^15^ are harsh, time-consuming, and, in some cases, highly localized, making them unfit for industrial-scale operations. The recent emergence of optical modification methods^16^ offers fast and simple alternatives that are easy to tune and upscale.

These predominantly laser-based methods leverage the energy of light to drive material property changes, such as doping,^17^ defect and phase engineering,^17−19^ and thinning.^20^ Understandably, most optical modification studies thus far have focused on modifying one 2D material at a time to introduce controlled and easily quantifiable changes.^16−20^ However, optical modification methods can be utilized to simultaneously modify multiple different 2D materials in a heterostructure^8^ due to their highly varying bond strengths^21^ and reactivity.^22^ This adds multifunctionality to the methods by enabling pathways and property-pairings otherwise nearly impossible to obtain and further cuts down fabrication time, highlighting the utility of this previously rather unexplored mechanism. For example, by applying the method to other 2D material pairings, optical modification could also be used to improve contact resistance of the full heterostructure stack^23^ or introduce doping from one constituent to another.^17^

In this paper, concurrent thinning of MoS_2_ and clustering of the Te atoms of deconstructed MoTe_2_ in a TMD heterostructure are demonstrated through optical modification utilizing a 532 nm continuous wave (CW) laser. The heterostructure is capped with hexagonal boron nitride (hBN) before it is exposed to the laser in order to insulate it from the environment and enhance the beneficial effects induced by the optical modification. These structural changes result in ∼43-fold PL enhancement in MoS_2_ and anti-ambipolar behavior in the MoTe_2_/MoS_2_ heterojunction. Additionally, a logic inverter with three logic states is demonstrated using the optically modified heterojunction devices. The changes are attributed to the thinning of MoS_2_ and changes in the dielectric environment caused by the tellurium clusters.

Multiple heterostructure samples with, in order from bottom-to-top, MoTe_2_ (1–4 layers (L)), MoS_2_ (2–4L), and hBN (∼25 nm) flakes are fabricated by mechanical exfoliation and transferred on top of each other with a micromanipulator tool. The samples are characterized with Raman and PL spectroscopies, and the flake thicknesses are estimated with low-frequency Raman measurements or optical contrast before they are optically modified using a 532 nm CW laser in a confocal Raman system. The important modification parameters: laser power, irradiation time, and separation between irradiation spots, are determined to be ∼15 mW, 2 s per spot, and 0.33 μm, respectively, and these parameters are used for all samples unless otherwise indicated. See Supporting Information Section 2 for more information on the parameter optimization and hBN capping.

The piezo stage of the confocal Raman system enables deterministic optical modification of heterostructures in complex patterns. As an example, the logo of Aalto University (A!) is patterned in a 2L MoTe_2_/2L MoS_2_ heterostructure. The structure is visible in both the bright-field and dark-field optical images presented in Figure 1a and b.

**Figure 1:**
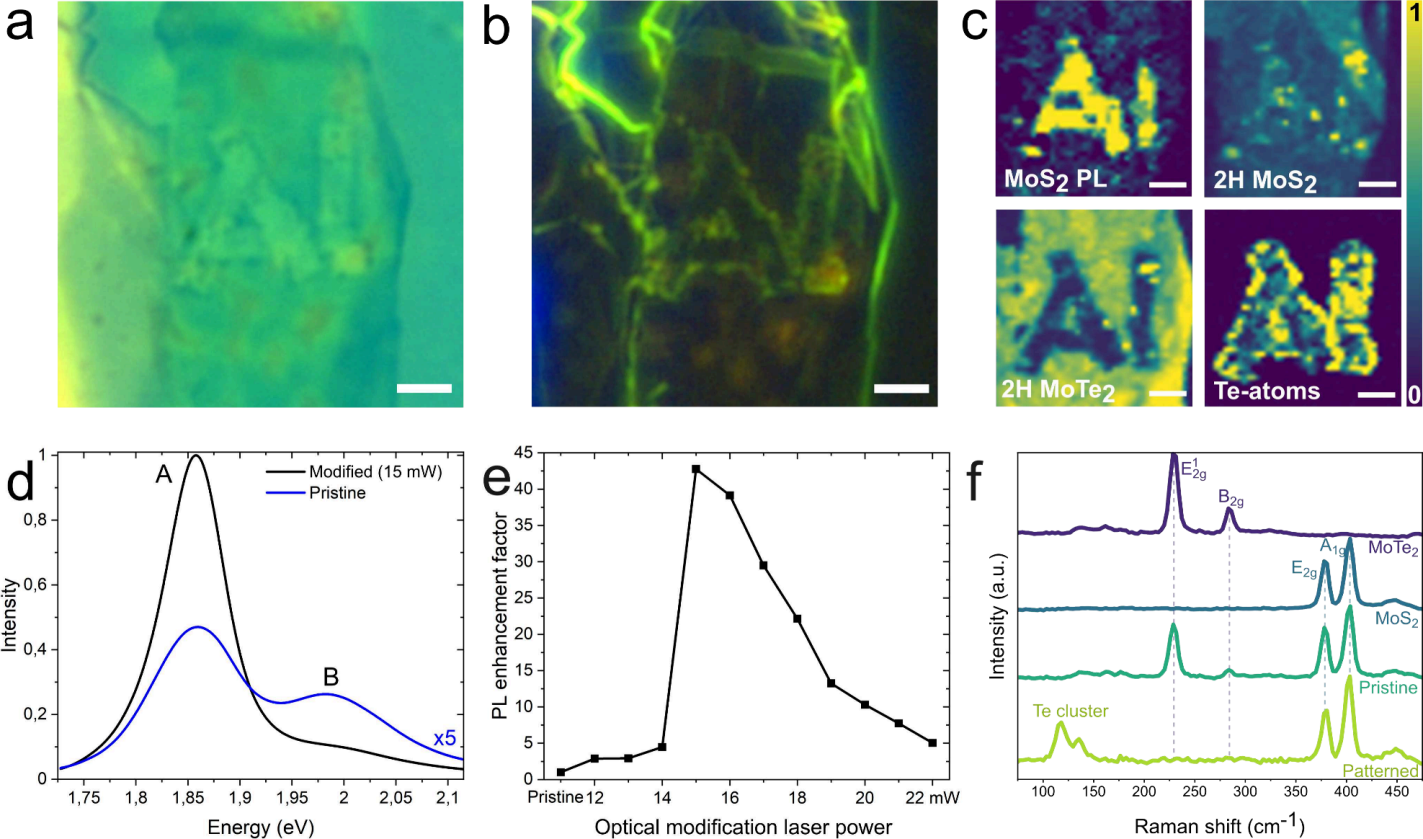
Optical images and characterization results of optically modified MoTe_2_/MoS_2_ heterostructures. **a)** Bright-field and **b)** dark-field optical images of optically modified Aalto University logo (A!) in the heterostructure. **c)** MoS_2_ PL map, 2H phase MoS_2_ (E_2*g*_), 2H phase MoTe_2_ (E_2g_^1^), and tellurium atom (∼120 cm^–1^) Raman maps. The scale bars in (a)–(c) are 2 μm. **d)** PL spectra of pristine and optically modified heterostructure area. The pristine spectrum has been multiplied by a factor of 5 for easier comparison. **e)** PL enhancement factors as a function of the optical modification laser powers. The points are connected by lines for easier viewability. **f)** Raman spectra of pristine MoTe_2_, MoS_2_, and heterostructure and optically modified heterostructure.

The Raman and PL mappings (Figure 1c) indicate several changes in the optical properties of the heterostructure after the optical modification. Perhaps most notably, the MoS_2_ PL response is enhanced in the optically modified area, a fact that becomes more evident in the PL spectra presented in Figure 1d and the optical modification laser power-dependent PL enhancement graph in Figure 1e. The full optical characterization of the power-dependence sample is presented in Supporting Information Figure S1.

Based on the results, the optical modification enhances the A-exciton contribution of the PL, reaching up to ∼43-fold PL enhancement with 15 mW optical modification laser power. The power-dependence graph shows that the PL is enhanced for all optical modification powers, with a high jump between 14 and 15 mW. The likely explanation for this is that below 15 mW, the laser merely anneals or defect-engineers^24^ the hBN-capped samples, while drastic structural changes occur above 15 mW, slowly becoming more detrimental until the PL enhancement is negligible with 22 mW optical modification laser power.

Indeed, significant MoS_2_ PL enhancement has been previously obtained with laser-driven thinning of air-exposed MoS_2_,^20^ changes in dielectric environment,^25,26^ strain,^27^ and defect engineering and doping.^24^ However, doping, strain, and changes in the dielectric environment typically also result in a shift or broadening of one or both PL peaks, which does not occur here in a significant manner (A-exciton shifts ∼2 meV and its full width at half-maximum (fwhm) broadens ∼35 meV). It should be noted that no PL enhancement is detected in optically modified MoS_2_ areas without the hBN capping (see Figure S2b), but this could also be due to the higher optical modification laser powers used here compared to the previous reports (2.5 mW).^20^

The possible structural transformations often manifest in changes in the Raman spectra (Figure 1f), and the modified MoS_2_ (2H phase) area does indeed show changes, albeit minor ones. The characteristic E_2g_ peak at ∼378 cm^–1^ shows a median blue-shift of ∼0.43 cm^–1^, broadening fwhm of ∼0.32 cm^–1^, and decrease in intensity by ∼3.5% across eight different samples. Similarly, the A_1g_ peak at ∼401 cm^–1^ exhibits a median blue-shift of ∼0.91 cm^–1^, narrows ∼0.24 cm^–1^, and increases in intensity by ∼1.7%. Additionally, the separation between the peaks increases by ∼0.69 cm^–1^. The origin of these changes is discussed later.

Although the changes to the MoS_2_ structure are minor, MoTe_2_ (2H phase) undergoes clear structural transformations in the optically modified areas. The characteristic MoTe_2_ 2H peaks at ∼229 cm^–1^ (E_2g_^1^) and ∼286 cm^–1^ (B_2g_) disappear completely, while a new peak at ∼120 cm^–1^ becomes very prominent. Previously, similar changes have been attributed to two separate causes: a phase change from 2H phase into 1T′ phase^19,28,29^ or clustering of the MoTe_2_ tellurium atoms.^30,31^ In our case, the Raman spectra of the optically modified area lack a relevant 1T′-phase peak (∼159 cm^–1^),^30^ indicating that the structural change is more likely due to the formation of metalloid tellurium clusters.

This conclusion is also supported by the cross-sectional transmission electron microscopy (TEM) and energy-dispersive X-ray spectroscopy (EDX) results. Figure 2a shows the structure of a pristine heterostructure with three distinct regions: silicon dioxide (SiO_2_), 2L MoTe_2_/2L MoS_2_ heterostructure (thickness confirmed optically, see Figure S2), and hBN. However, in the TEM image of the optically modified area (Figure 2b), the heterostructure is almost completely destroyed, leaving only a thin MoS_2_ layer at the very top and a new dark feature embedded in SiO_2_. Based on the EDX results (Figure 2c), these darker areas consist of silicon, oxygen, tellurium, and molybdenum. This indicates that the optical modification partially implants elements from the heterostructure, especially from the MoTe_2_ layer, into the SiO_2_. Additionally, the embedded materials show evidence of crystallinity (Figure S3). Based on stoichiometry, the crystalline regions are estimated to consist of oxygen degraded silicon telluride (Si_2_Te_2_),^32^ which also has a prominent Raman peak at ∼120 cm^–1^ and a smaller peak at ∼140 cm^–1^, just as observed in this paper. It should also be noted that the oxygen concentration in hBN is most likely an error from the close proximity of the N and O peaks, but it could also be from slight oxygen doping of hBN.^33^

**Figure 2:**
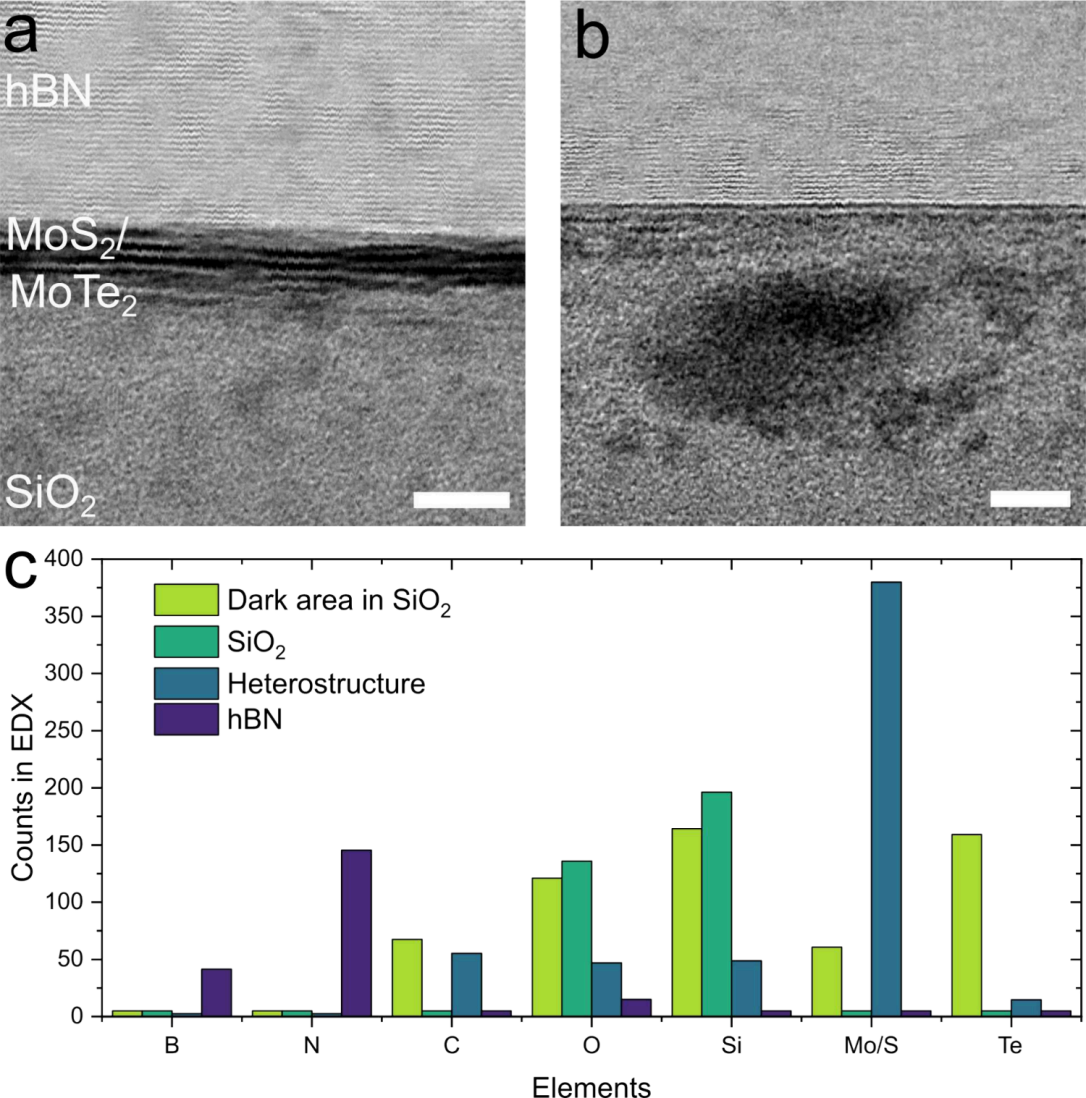
Transmission electron microscopy (TEM) and energy dispersive X-ray (EDX) spectroscopy results of the pristine and optically modified heterostructure. **a)** TEM image of the pristine heterostructure; **b)** TEM image of the optically modified heterostructure. The scale bars in the TEM images are 5 nm. **c)** EDX analysis of the dark feature in SiO_2_, SiO_2_ heterostructure, and hBN.

The complete deconstruction of MoTe_2_ compared to the simple thinning of the MoS_2_ is logical, as the bond strength in MoTe_2_ is weaker than in MoS_2_.^21^ The TEM results are also supported by the disappearance of low-frequency Raman peaks in the optically modified heterostructure (Figure S4e), which indicates a lack of interactions between layers. These findings can also explain both the observed PL enhancement and the MoTe_2_ Raman spectrum changes. However, they cannot explain the shifting of the MoS_2_ Raman peaks nor their widening separation, as the peaks should shift closer to each other upon thinning.^34^

The shifting of the MoS_2_ Raman peaks could be attributed to the changes in the dielectric environment^25,26^ or doping.^35,36^ It has been shown previously that an environment with a relative permittivity (κ) between 15 and 20 can broaden the MoS_2_ E_2*g*_ peak and lead to a larger separation between the E_2*g*_ and A_1*g*_ peaks, while also having higher PL intensity and a comparatively similar peak position to MoS_2_ on SiO_2_, which has a relative permittivity^37^ of ∼3.9.^26^ As tellurium has a much higher relative permittivity (∼27.5 for polycrystalline^38^), it could locally modify the dielectric environment to this range, explaining the observed changes. On the other hand, tellurium doping (p-type) of MoS_2_ has been shown to induce blue-shifting of both Raman peaks, fitting these results, but also significant shifting and intensity decrease of PL,^35^ neither of which are observed here. This led us to conclude that the samples exhibit no significant structural doping.

To evaluate the effect of optical modification on the electrical properties and the surface potential of heterostructures, a 3L MoTe_2_/3L MoS_2_ heterojunction transistor (Figure 3a and b) is designed and fabricated for optical modification. The optical characterization of the junction, presented in Figure S4, indicates a clean heterointerface but, unlike all the other samples, does not display PL enhancement. This is likely due to the polymer residues left from the electrode patterning process. See more experimental evidence and discussion on the topic in the Supporting Information.

**Figure 3:**
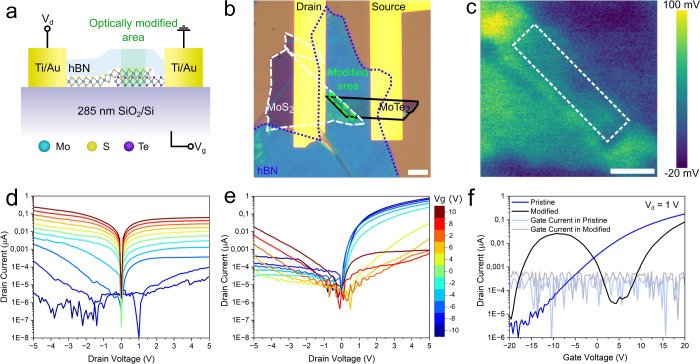
Electrical characterization of a MoTe_2_/MoS_2_ heterojunction. **a)** Schematic illustration of the optical modification process applied to the junction area. **b)** Optical image of the heterojunction. The scale bar is 5 μm. **c)** Kelvin probe force microscopy (KPFM) potential map of the optically modified MoTe_2_/MoS_2_ heterostructure. The scale bar is 2 μm, and the white dashed box indicates the optically modified area. **d)** The output curve of the pristine MoTe_2_/MoS_2_ heterojunction, **e)** the output curve of the optically modified MoTe_2_/MoS_2_ heterojunction, and **f)** transfer curves of the heterojunction before and after optical modification for *V*_*d*_ = 1 V. Gate currents are included to illustrate that leakage has no effect on the curves.

The Kelvin probe force microscopy (KPFM) surface potential map (Figure 3c) and graph (Figure S5b) indicate that there is a decrease in the surface potential in the optically modified heterojunction. Previously a similar phenomenon has been observed for MoS_2_ on a high-κ substrate due to the dielectric screening effect^39^ and with doping due to changes in carrier concentrations.^40^

More information about the observed surface potential changes can be gained from the output characteristics in Figure 3d and e, which originally exhibit symmetric and monotonically gate-dependent responses but become asymmetric after optical modification, also displaying gate-dependent rectifying behavior. Drastic changes are also seen in the transfer characteristics, as presented in Figure 3f. Prior to optical modification, the device exhibits a monotonic n-type transfer curve at a drain voltage *V*_*d*_ = 1 V, but after optical modification, the transfer curve transforms into a bell-shaped response when the gate voltage (*V*_*g*_) is swept from −20 to 4 V, indicating a transition from monotonic to nonmonotonic, anti-ambipolar behavior. Additionally, the transconductance (g_*m*_ = ), plotted in Figure S7, displays negative values between *V*_*g*_ = −8 and *V*_*g*_ = 4 V.

The observed changes could be attributed to many different phenomena, including band-alignment changes,^8^ conductive bridges of the tellurium clusters,^41^ doping,^42,43^ and dielectric screening.^9^ Before optical modification, the MoTe_2_/MoS_2_ heterojunction exhibits weaker gate tunability, resulting in n-type characteristics similar to those observed in individual MoTe_2_ or MoTe_2_ transistors.^23,44^ The modification process thins the MoS_2_ and MoTe_2_ layers and strengthens the gate-controlled band evolution.^45^ Based on the results, especially the changes in Raman and PL, we speculate that the most likely causes of the aforementioned changes are band alignment and work function adjustment induced by the thinning of MoS_2_^46^ and a dielectric screening effect^9^ from the tellurium clusters embedded into the SiO_2_.

Finally, a similar optically modified MoTe_2_/MoS_2_ heterojunction (see Figures S6 and S8 for optical and electrical characterization, and Figure S7 for transconductance) is connected to an external resistor (120 MΩ) to demonstrate the optically modified heterojunction’s performance as an inverter, and the results are presented in Figure 4. As expected from the anomalous transfer curve, the output voltage (*V*_*out*_) experiences a voltage at the drain (*V*_*DD*_)-dependent dip below *V*_*g*_ = −10 V (Figure 4a), which manifests as a second feature in the voltage gain  graph (Figure 4b). This indicates that the device is showing ternary inverting properties with the logic states first at below *V*_*g*_ = −10 V, then between *V*_*g*_ = −10 V and *V*_*g*_ = ∼5 V, and last above *V*_*g*_ = ∼5 V. The gain of the logic state above *V*_*g*_ = ∼5 V is reasonably over 1 (for *V*_*DD*_ = 5 V), which is a requirement for use in practical applications;^47,48^ however, the first gain feature only reaches up to 0.4. Nevertheless, the results indicate these optically modified heterojunctions could act as efficient multivalue logic inverters for applications especially after optimizing the modification parameters, device materials,^49,50^ dimensions,^45,51^ and fabrication cleanliness.^52−54^ For example, optimization could enable tunable junctions capable of achieving high spectral resolution and broad operational bandwidth for devices in spectrometers.^13,55^ Additionally, more device data could also be acquired with different electrode configurations^49,56^ or materials.^50,52^

**Figure 4:**
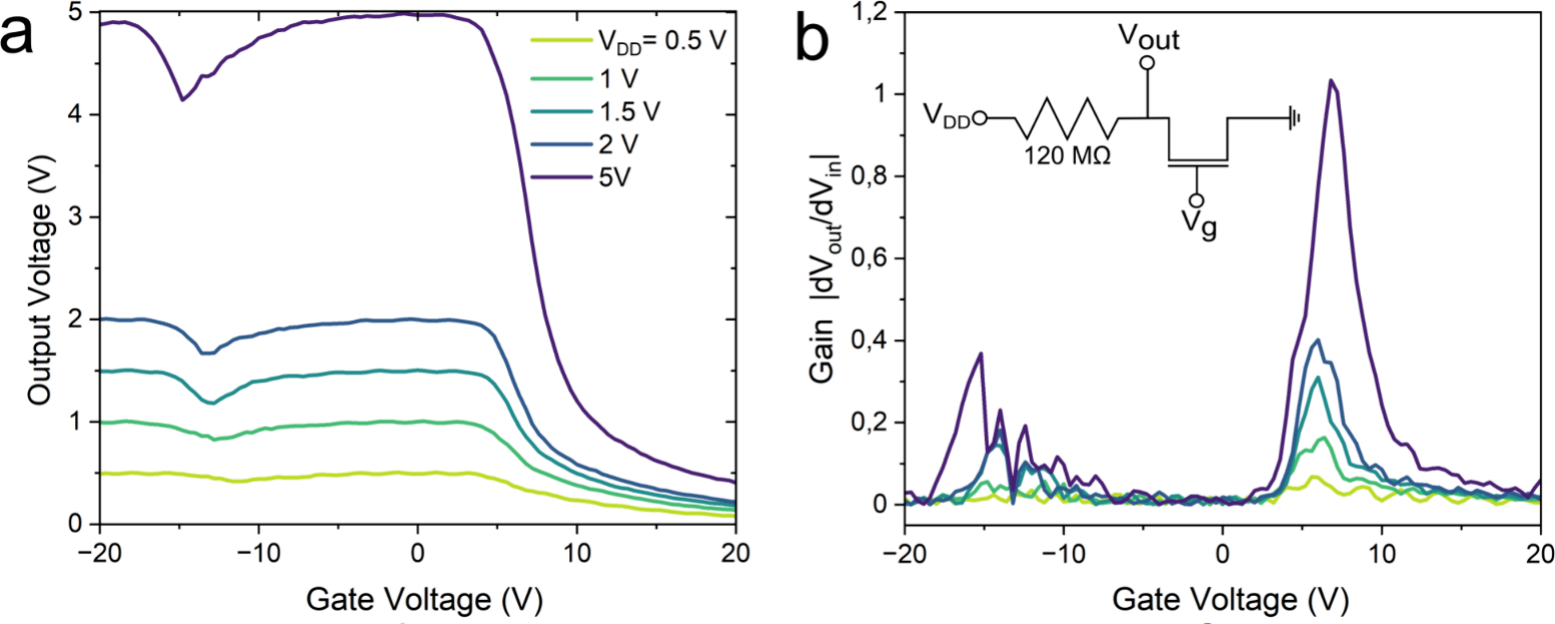
Performance of the optically modified heterojunction as a logic inverter. **a)** Voltage transfer characteristic curve  of the modified heterojunction and **b)** voltage gain graph of the modified heterojunction. The measurement circuit is presented in the inset.

In summary, optical modification of hBN-capped MoTe_2_/MoS_2_ heterostructures results in a ∼43-fold MoS_2_ PL enhancement and anti-ambipolar electric behavior. The optical modification induces thinning of MoS_2_ and deconstruction and clustering of tellurium atoms of MoTe_2_, as confirmed by TEM measurements. These structural transformations lead to changes in the dielectric environment and band alignment, explaining the observed results. The optically modified heterojunction is also demonstrated as an inverter with three logic states. The observed results highlight the potential of heterostructure optical modification for applications in novel (opto)electronic devices, including logic circuits and spectrometers.

## Supporting Information Available

Additional experimental details, characterization methods, and figures presenting Raman, AFM, STM, and electrical measurement results (PDF)

## References

[ref1] Geim A. K.; Novoselov K. S. The rise of graphene. Nat. Mater. 2007, 6, 183–191. 10.1038/nmat1849. 17330084

[ref2] Manzeli S.; Ovchinnikov D.; Pasquier D.; Yazyev O. V.; Kis A. 2D transition metal dichalcogenides. Nature Reviews Materials 2017, 2, 17033 10.1038/natrevmats.2017.33.

[ref3] Splendiani A.; Sun L.; Zhang Y.; Li T.; Kim J.; Chim C. Y.; Galli G.; Wang F. Emerging photoluminescence in monolayer MoS_2_. Nano Lett. 2010, 10, 1271–1275. 10.1021/nl903868w. 20229981

[ref4] Chaves A.; et al. Bandgap engineering of two-dimensional semiconductor materials. npj 2D Materials and Applications 2020, 4, 1–21. 10.1038/s41699-020-00162-4.

[ref5] Gong C.; Zhang Y.; Chen W.; Chu J.; Lei T.; Pu J.; Dai L.; Wu C.; Cheng Y.; Zhai T.; Li L.; Xiong J. Electronic and Optoelectronic Applications Based on 2D Novel Anisotropic Transition Metal Dichalcogenides. Advanced Science 2017, 4, 1700231 10.1002/advs.201700231. 29270337 PMC5737141

[ref6] Jiang Y.; Chen S.; Zheng W.; Zheng B.; Pan A. Interlayer exciton formation, relaxation, and transport in TMD van der Waals heterostructures. Light: Science Applications 2021, 10, 1–29. 10.1038/s41377-021-00500-1. 33811214 PMC8018964

[ref7] Li C.; Yan X.; Song X.; Bao W.; Ding S.; Zhang D. W.; Zhou P. WSe_2_/MoS_2_ and MoTe_2_/SnSe_2_ van der Waals heterostructure transistors with different band alignment. Nanotechnology 2017, 28, 415201 10.1088/1361-6528/aa810f. 28726689

[ref8] Huo J.; Zou G.; Xiao Y.; Sun T.; Feng B.; Shen D.; Du C.; Peng J.; Lin L.; Liu L. Multifunctional van der Waals heterostructures enabled by femtosecond laser-controlled band alignment engineering. Nano Energy 2023, 113, 108548 10.1016/j.nanoen.2023.108548.

[ref9] Geng G.; Wu E.; Xu L.; Hu X.; Miao X.; Zou J.; Wu S.; Liu J.; Liu Y.; He Z. Dielectric engineering enable to lateral anti-ambipolar MoTe_2_ heterojunction. Nanotechnology 2022, 33, 175704 10.1088/1361-6528/ac49c2. 35008081

[ref10] Liu C.; Chen H.; Wang S.; Liu Q.; Jiang Y. G.; Zhang D. W.; Liu M.; Zhou P. Two-dimensional materials for next-generation computing technologies. Nat. Nanotechnol. 2020, 15, 545–557. 10.1038/s41565-020-0724-3. 32647168

[ref11] Liu C.; Yan X.; Song X.; Ding S.; Zhang D. W.; Zhou P. A semi-floating gate memory based on van der Waals heterostructures for quasi-non-volatile applications. Nat. Nanotechnol. 2018, 13, 404–410. 10.1038/s41565-018-0102-6. 29632398

[ref12] Pham P. V.; Bodepudi S. C.; Shehzad K.; Liu Y.; Xu Y.; Yu B.; Duan X. 2D Heterostructures for Ubiquitous Electronics and Optoelectronics: Principles, Opportunities, and Challenges. Chem. Rev. 2022, 122, 6514–6613. 10.1021/acs.chemrev.1c00735. 35133801

[ref13] Yoon H. H.; Fernandez H. A.; Nigmatulin F.; Cai W.; Yang Z.; Cui H.; Ahmed F.; Cui X.; Uddin M. G.; Minot E. D.; Lipsanen H.; Kim K.; Hakonen P.; Hasan T.; Sun Z. Miniaturized spectrometers with a tunable van der Waals junction. Science 2022, 378, 296–299. 10.1126/science.add8544. 36264793

[ref14] Wu G.; Abid M.; Zerara M.; Cho J.; Choi M.; Coileáin C.; Hung K.-M.; Chang C.-R.; Shvets I. V.; Wu H.-C. Miniaturized spectrometer with intrinsic long-term image memory. Nat. Commun. 2024, 15, 676 10.1038/s41467-024-44884-1. 38263315 PMC10805890

[ref15] Kirubasankar B.; Won Y. S.; Adofo L. A.; Choi S. H.; Kim S. M.; Kim K. K. Atomic and structural modifications of two-dimensional transition metal dichalcogenides for various advanced applications. Chemical Science 2022, 13, 7707–7738. 10.1039/D2SC01398C. 35865881 PMC9258346

[ref16] Akkanen S. M.; Fernandez H. A.; Sun Z. Optical Modification of 2D Materials: Methods and Applications. Adv. Mater. 2022, 34, 2110152 10.1002/adma.202110152. 35139583

[ref17] Kim E.; Ko C.; Kim K.; Chen Y.; Suh J.; Ryu S.-G.; Wu K.; Meng X.; Suslu A.; Tongay S.; Wu J.; Grigoropoulos C. P. Site Selective Doping of Ultrathin Metal Dichalcogenides by Laser-Assisted Reaction. Adv. Mater. 2016, 28, 341–346. 10.1002/adma.201503945. 26567761

[ref18] Akkanen S. T. M.; Arias-Muñoz J. C.; Emelianov A. V.; Mentel K. K.; Tammela J. V.; Partanen M.; Das S.; Faisal A.; Pettersson M.; Sun Z. Enhanced Nonlinear Optical Responses in MoS_2_ via Femtosecond Laser-Induced Defect-Engineering. Adv. Funct. Mater. 2024, 34, 2406942 10.1002/adfm.202406942.

[ref19] Ahmed F.; et al. Deterministic Polymorphic Engineering of MoTe_2_ for Photonic and Optoelectronic Applications. Adv. Funct. Mater. 2023, 33, 2302051 10.1002/adfm.202302051.

[ref20] Hu L.; Shan X.; Wu Y.; Zhao J.; Lu X. Laser Thinning and Patterning of MoS_2_ with Layer-by-Layer Precision. Sci. Rep. 2017, 7, 15538 10.1038/s41598-017-15350-4. 29138427 PMC5686209

[ref21] Tan Y.; Bo M. Electrostatic shielding effects and binding energy shifts and topological phases of bilayer molybdenum chalcogenides. ChemistrySelect 2024, 9, e202303817 10.1002/slct.202303817.

[ref22] Politano A.; Vitiello M. S.; Viti L.; Boukhvalov D. W.; Chiarello G. The role of surface chemical reactivity in the stability of electronic nanodevices based on two-dimensional materials “beyond graphene” and topological insulators. FlatChem. 2017, 1, 60–64. 10.1016/j.flatc.2016.11.003.

[ref23] Mleczko M. J.; Yu A. C.; Smyth C. M.; Chen V.; Shin Y. C.; Chatterjee S.; Tsai Y. C.; Nishi Y.; Wallace R. M.; Pop E. Contact Engineering High-Performance n-Type MoTe2 Transistors. Nano Lett. 2019, 19, 6352–6362. 10.1021/acs.nanolett.9b02497. 31314531

[ref24] Nan H.; Wang Z.; Wang W.; Liang Z.; Lu Y.; Chen Q.; He D.; Tan P.; Miao F.; Wang X.; Wang J.; Ni Z. Strong photoluminescence enhancement of MoS_2_ through defect engineering and oxygen bonding. ACS Nano 2014, 8, 5738–5745. 10.1021/nn500532f. 24836121

[ref25] Buscema M.; Steele G. A.; van der Zant H. S.; Castellanos-Gomez A. The effect of the substrate on the Raman and photoluminescence emission of single-layer MoS_2_. Nano Research 2014, 7, 561–571. 10.1007/s12274-014-0424-0.

[ref26] Lin Y.; Ling X.; Yu L.; Huang S.; Hsu A. L.; Lee Y. H.; Kong J.; Dresselhaus M. S.; Palacios T. Dielectric screening of excitons and trions in single-layer MoS_2_. Nano Lett. 2014, 14, 5569–5576. 10.1021/nl501988y. 25216267

[ref27] Dhakal K. P.; Roy S.; Jang H.; Chen X.; Yun W. S.; Kim H.; Lee J.; Kim J.; Ahn J. H. Local Strain Induced Band Gap Modulation and Photoluminescence Enhancement of Multilayer Transition Metal Dichalcogenides. Chem. Mater. 2017, 29, 5124–5133. 10.1021/acs.chemmater.7b00453.

[ref28] Cho S.; Kim S.; Kim J. H.; Zhao J.; Seok J.; Keum D. H.; Baik J.; Choe D.-H.; Chang K. J.; Suenaga K.; Kim S. W.; Lee Y. H.; Yang H. Phase patterning for ohmic homojunction contact in MoTe_2_. Science 2015, 349, 625–628. 10.1126/science.aab3175. 26250680

[ref29] Tan Y.; Luo F.; Zhu M.; Xu X.; Ye Y.; Li B.; Wang G.; Luo W.; Zheng X.; Wu N.; Yu Y.; Qin S.; Zhang X. A. Controllable 2H-to-1T phase transition in few-layer MoTe_2_. Nanoscale 2018, 10, 19964–19971. 10.1039/C8NR06115G. 30349910

[ref30] Kowalczyk H.; Biscaras J.; Pistawala N.; Harnagea L.; Singh S.; Shukla A. Gate and Temperature Driven Phase Transitions in Few-Layer MoTe_2_. ACS Nano 2023, 17, 6708–6718. 10.1021/acsnano.2c12610. 36972180

[ref31] Zakhidov D.; Rehn D. A.; Reed E. J.; Salleo A. Reversible Electrochemical Phase Change in Monolayer to Bulk-like MoTe_2_ by Ionic Liquid Gating. ACS Nano 2020, 14, 2894–2903. 10.1021/acsnano.9b07095. 32045212

[ref32] Hathaway E.; Rodriguez R. G.; Lin Y.; Cui J. Elucidating the Degradation Mechanisms in Silicon Telluride through Multimodal Characterization. J. Phys. Chem. C 2023, 127, 13800–13809. 10.1021/acs.jpcc.3c01864.

[ref33] Liu K.; Zhu X.; Lin B.; Lu Z.; Zhang G. Effect of oxygen atoms adsorption and doping on hexagonal boron nitride. Physica E: Low-dimensional Systems and Nanostructures 2022, 135, 114977 10.1016/j.physe.2021.114977.

[ref34] Molina-Sánchez A.; Hummer K.; Wirtz L. Vibrational and optical properties of MoS_2_: From monolayer to bulk. Surf. Sci. Rep. 2015, 70, 554–586. 10.1016/j.surfrep.2015.10.001.

[ref35] Oh G. H.; il Kim S.; Kim T. W. High-performance Te-doped p-type MoS_2_ transistor with high-K insulators. J. Alloys Compd. 2021, 860, 157901 10.1016/j.jallcom.2020.157901.

[ref36] Iqbal M. W.; Shahzad K.; Akbar R.; Hussain G. A review on Raman finger prints of doping and strain effect in TMDCs. Microelectron. Eng. 2020, 219, 111152 10.1016/j.mee.2019.111152.

[ref37] Kazimierczuk M. K.; Ayachit A. Pulse-Width Modulated DC-DC Power Converters, 2nd ed.; John Wiley and Sons, Ltd., 2016; p 915.

[ref38] Young K. F.; Frederikse H. P. Compilation of the Static Dielectric Constant of Inorganic Solids. J. Phys. Chem. Ref. Data 1973, 2, 313–410. 10.1063/1.3253121.

[ref39] Ouyang Y.; Jiang Z.; Ulstrup S.; Guo Z.; Wang Z.; Dong M. Enhancing MoS_2_ Electronic Performance with Solid-State Lithium-Ion Electrolyte Contacts through Dielectric Screening. ACS Nano 2024, 18, 33310–33318. 10.1021/acsnano.4c05973. 39611299

[ref40] Kim Y.; Bark H.; Kang B.; Lee C. Wafer-Scale Substitutional Doping of Monolayer MoS_2_ Films for High-Performance Optoelectronic Devices. ACS Appl. Mater. Interfaces 2019, 11, 12613–12621. 10.1021/acsami.8b20714. 30873829

[ref41] Lee M.; Kim T. W.; Park C. Y.; Lee K.; Taniguchi T.; Watanabe K.; gu Kim M.; Hwang D. K.; Lee Y. T. Graphene Bridge Heterostructure Devices for Negative Differential Transconductance Circuit Applications. Nano-Micro Letters 2023, 15, 1–11. 10.1007/s40820-022-01001-5. PMC9800667 36580180

[ref42] Wu E.; Xie Y.; Liu Q.; Hu X.; Liu J.; Zhang D.; Zhou C. Photoinduced Doping to Enable Tunable and High-Performance Anti-Ambipolar MoTe_2_/MoS_2_ Heterotransistors. ACS Nano 2019, 13, 5430–5438. 10.1021/acsnano.9b00201. 30974935

[ref43] Hu R.; Wu E.; Xie Y.; Liu J. Multifunctional anti-ambipolar p–n junction based on MoTe_2_/MoS_2_ heterostructure. Appl. Phys. Lett. 2019, 115, 073104 10.1063/1.5109221.

[ref44] Pang Y.; Zhou Y.; Tong L.; Xu J. 2D Dual Gate Field-Effect Transistor Enabled Versatile Functions. Small 2024, 20, 2304173 10.1002/smll.202304173. 37705128

[ref45] Duong N. T.; Lee J.; Bang S.; Park C.; Lim S. C.; Jeong M. S. Modulating the Functions of MoS2/MoTe2 van der Waals Heterostructure via Thickness Variation. ACS Nano 2019, 13, 4478–4485. 10.1021/acsnano.9b00014. 30938981

[ref46] Lin M. W.; Kravchenko I. I.; Fowlkes J.; Li X.; Puretzky A. A.; Rouleau C. M.; Geohegan D. B.; Xiao K. Thickness-dependent charge transport in few-layer MoS_2_ field-effect transistors. Nanotechnology 2016, 27, 165203 10.1088/0957-4484/27/16/165203. 26963583

[ref47] Radisavljevic B.; Whitwick M. B.; Kis A. Integrated Circuits and Logic Operations Based on Single-Layer MoS_2_. ACS Nano 2011, 5, 9934–9938. 10.1021/nn203715c. 22073905

[ref48] Das S.; Dubey M.; Roelofs A. High gain, low noise, fully complementary logic inverter based on bi-layer WSe_2_ field effect transistors. Appl. Phys. Lett. 2014, 105, 083511 10.1063/1.4894426.

[ref49] Sun Y.; Gao W.; Li X.; Xia C.; Chen H.; Zhang L.; Luo D.; Fan W.; Huo N.; Li J. Anti-ambipolar behavior and photovoltaic effect in p-MoTe2/n-InSe heterojunctions. Journal of Materials Chemistry C 2021, 9, 10372–10380. 10.1039/D1TC02497C.

[ref50] Huang M.; Li S.; Zhang Z.; Xiong X.; Li X.; Wu Y. Multifunctional high-performance van der Waals heterostructures. Nature Nanotechnology 2017 12:12 2017, 12, 1148–1154. 10.1038/nnano.2017.208. 28991241

[ref51] Chhowalla M.; Jena D.; Zhang H. Two-dimensional semiconductors for transistors. Nature Reviews Materials 2016 1:11 2016, 1, 1–15. 10.1038/natrevmats.2016.52.

[ref52] Yao H.; Wu E.; Liu J. Frequency doubler based on a single MoTe2/MoS2anti-ambipolar heterostructure. Appl. Phys. Lett. 2020, 117, 123103 10.1063/5.0018882.

[ref53] Lee J. H.; Lee H. B.; Jeong N. B.; Park D. H.; Choi I.; Chung H. J. High-speed residue-free transfer of two-dimensional materials using PDMS stamp and water infiltration. Curr. Appl. Phys. 2020, 20, 1190–1194. 10.1016/j.cap.2020.07.019.

[ref54] Jain A.; Bharadwaj P.; Heeg S.; Parzefall M.; Taniguchi T.; Watanabe K.; Novotny L. Minimizing residues and strain in 2D materials transferred from PDMS. Nanotechnology 2018, 29, 265203 10.1088/1361-6528/aabd90. 29644983

[ref55] Deng W.; et al. Electrically tunable two-dimensional heterojunctions for miniaturized near-infrared spectrometers. Nature Communications 2022 13:1 2022, 13, 1–10. 10.1038/s41467-022-32306-z. PMC9360404 35941126

[ref56] Zhang S.; Le S. T.; Richter C. A.; Hacker C. A. Improved contacts to p-type MoS2 transistors by charge-transfer doping and contact engineering. Appl. Phys. Lett. 2019, 115, 73106 10.1063/1.5100154. PMC7047721 32116333

