# Supplementary material for: Optical Modification of TMD Heterostructures
Source: Nano Lett. 2025 Mar 5;25(11):4379–85. doi: 10.1021/acs.nanolett.4c06512 (PMC11926954; doi:10.1021/acs.nanolett.4c06512)
Supplement: Supplementary file 1 — nl4c06512_si_001.pdf [file nl4c06512_si_001.pdf]

Optical modification of TMD heterostructures

Suvi-Tuuli Varjamo,* Christopher Edwards, Yaoqiang Zhou, Ruihuan Fang, Seyed Hossein Hosseini Shokouh, and Zhipei Sun*

Department of Electronics and Nanoengineering, Aalto University, Espoo 02150, Finland

E-mail: suvi-tuuli.varjamo@aalto.fi; zhipei.sun@aalto.fi

Experimental details

Sample fabrication

The samples are fabricated by mechanical exfoliation of bulk 2D $MoTe_2$, MoS_2, and hBN crystals (2D semiconductors) using the scotch-tape method, first with Nitto tape and later with polydimethyl siloxane (PDMS). The thicknesses of the flakes are estimated by utilizing optical contrast between flakes. The flakes are then stacked on top of each other on 285 nm silicon/silicon oxide substrates using an in-house built micromanipulation setup and a microscope. The sample stage is heated to 60 °C during transfer.

To fabricate heterojunction devices using stacks fabricated as above, electrodes are patterned on polymethyl methacrylate photoresist by utilizing electron beam lithography (Vistec EPBG5000pES). 5 nm titanium and 50 nm gold are deposited in the patterns through physical vapor deposition (Angstrom Engineering).

Optical modification and parameter optimization

The samples are optically modified via laser patterning with a commercial WiTec Alpha RA+ Confocal Raman setup with a 532 nm CW laser and Nikon 100x CF Plan (NA=0.95)

objective. The patterning is done with the tool's image scan mode, which utilizes a raster pattern with a resolution determined by lines per image and points per line.

The optical modification parameters were initially optimized with the new $MoTe_2$-related Raman peaks at 120 and 140 cm^{-1} in mind as they have previously been attributed to 2H-to-1T' phase change[1] or formation of tellurium clusters,[2] as is the case here. The testing was first done for uncovered 1-2L $MoTe_2$ flakes with different wavelengths and powers (See Table S1) as the phenomenon has not yet been successfully demonstrated for such thin flakes. As the results were not promising, the process was repeated with hBN covering using a 532 nm laser with different powers, irradiation times and step sizes. The results, presented in table S2, show that the most optimal parameters for the structural changes behind the introduction of new Raman peaks are 15 mW power, 2 s irradiation time and 0.33 μm separation, although the gradient sample in Table S3 also shows good results with some higher powers. The parameters for highest MoS2 PL enhancement through optically induced thinning were then optimized by changing only the power as shown in Table S3. Based on the results, the optimal conditions for PL enhancement is achieved with 15 mW modification power, 2 s irradiation time and 0.33 µm spot separation.

The inclusion of hBN has many benefits. As an insulating material it acts as a heat barrier, filtering out a lot of the optical power before it reaches the sample, ensuring gentler patterning. Additionally, it acts as a barrier between ambient molecules and surface impurities that could otherwise disturb the patterning process or lead to unwanted phenomena such as oxidation.[3,4]

Table S1: Optical modification of Bare $MoTe_2$ under different laser wavelengths and powers.

Power (mW)	Raman peak intensity compared to pristine (%)		
	$I(E^1_{2g})$	$I(B_{2g})$	I(Te-peak)
**532 nm laser, 0.1 s irradiation time, 0.33 µm separation**			
4	64.3	34.4	52.7
5	40.3	20.3	58.1
6	24.0	17.4	51.8
8	6.9	9.5	29.3
10	5.0	11.3	27.9
**488 nm laser, 0.1 s irradiation time, 0.33 µm separation**			
4	55.4	27.1	91.6
5	38.4	20.1	78.5
6	28.3	23.3	93.2
8	13.7	17.2	68.1
10	6.5	9.5	40.3
**633 nm laser, 0.1 s irradiation time, 0.33 µm separation**			
4	72.6	74.9	74.3
5	63.6	28.9	99.0
6	57.5	20.4	132.5
8	38.3	17.9	86.9
10	20.6	12.0	77.5

Table S2: Optical modification of hBN capped $MoTe_2$ under different powers, irradiation time, and spot separation. The laser wavelength is 532 nm.

**Power** (mW)	**Integration** time (s)	**Step** size (μm)	**Raman and PL peak intensity compared to pristine (%)** E_{2g}^1 ($MoTe_2$)	B_{2g} ($MoTe_2$)	Te
12.5	2	0.33	100.2	93.6	99.7
12.5	2	0.5	99.9	94.4	99.7
15	2	0.33	102.8	96.5	156.7
15	3	0.33	99.8	94.4	99.6
15	5	0.33	99.8	94.3	99.5
16.25	2	0.33	100.0	95.0	99.7
16.5	2	0.33	99.5	91.6	96.9
16.5	3	0.33	99.9	82.6	95.0
16.5	2	0.5	98.3	86.6	95.4
16.5	3	1	97.3	85.3	94.9
16.5	5	1	97.0	92.8	95.8
16.5	2	1	97.2	96.8	95.8
17	2	0.33	102.4	100.6	102.3
17	0.2	0.5	100.0	98.8	100.0
20	0.5	0.1	99.9	98.5	99.9

Table S3: Optical modification of hBN capped heterostructures under different powers, irradiation times and spot separation. The laser wavelength is 532 nm.

**Power (mW)**	**Raman and PL peak intensity compared to pristine (%)**					
	**$MoTe_2$**			**MoS_2**		
	Te	E^1_{2g}	B_{2g}	E_{2g}	A_{1g}	PL
12	387.6	5.3	22.6	91.6	92.6	287.6
13	348.4	5.8	26.0	103.3	109.2	292.1
14	385.7	5.7	36.7	102.0	110.1	580.2
15	510.2	8.5	51.5	49.0	60.0	6082.6
16	348.6	7.3	45.0	64.8	72.9	5226.2
17	378.1	6.8	43.7	73.1	88.3	3952.6
18	397.4	6.3	55.6	69.0	84.4	2927.0
19	1127.3	8.9	53.0	70.8	83.9	1765.0
20	761.8	8.4	51.5	72.5	86.5	1360.7
21	834.5	10.7	51.9	70.0	72.0	969.1
22	2106.3	10.4	63.4	95.8	101.9	627.8

Raman, Low-Frequency Raman and Photoluminescence characterization

All optical spectroscopy methods are conducted on the same WiTec Alpha RA+ Confocal Raman setup as the patterning with the same wavelength and objective. The grating used for PL and Raman measurements is 600 g/mm. For low-frequency Raman mode, the built-in 532 nm filter is removed and Bragg-grating filters are engaged. The low-frequency Raman measurement is conducted with a 1800 g/mm grating. All of the measurements are performed with 700 μW power.

Atomic Force Microscopy (AFM) and Kelvin Probe Force Microscopy (KPFM)

The force microscopy methods are performed using a Bruker Dimension Icon tool. Height analysis with AFM is conducted in ScanAsyst air mode with a ScanAsyst air tip and KPFM

measurements are conducted in electrical measurement mode with a PFTUNA tip.

Cross-sectional transmission electron microscopy (TEM) and electron dispersive X-ray spectroscopy (EDX)

The sample is coated with 20 nm platinum via physical vapor deposition (Angstrom engineering) to prepare it for lamella-making (JEOL JIB-4700F). TEM and EDX are performed with JEOL JEM-2200 FS. See a more detailed description in ref.[5]

Electrical measurements

The electrical measurements are performed using a semiconductor device parameter analyzer (Keysight B1500A) and a probe station (Everbeing Int'l Corp, C-Series). The gate is applied through the Si/SiO_2 substrate. The external resistor utilized in the inverter measurements is an electric breadboard with ten 15 MΩ resistors connected in series.

Supporting Figures

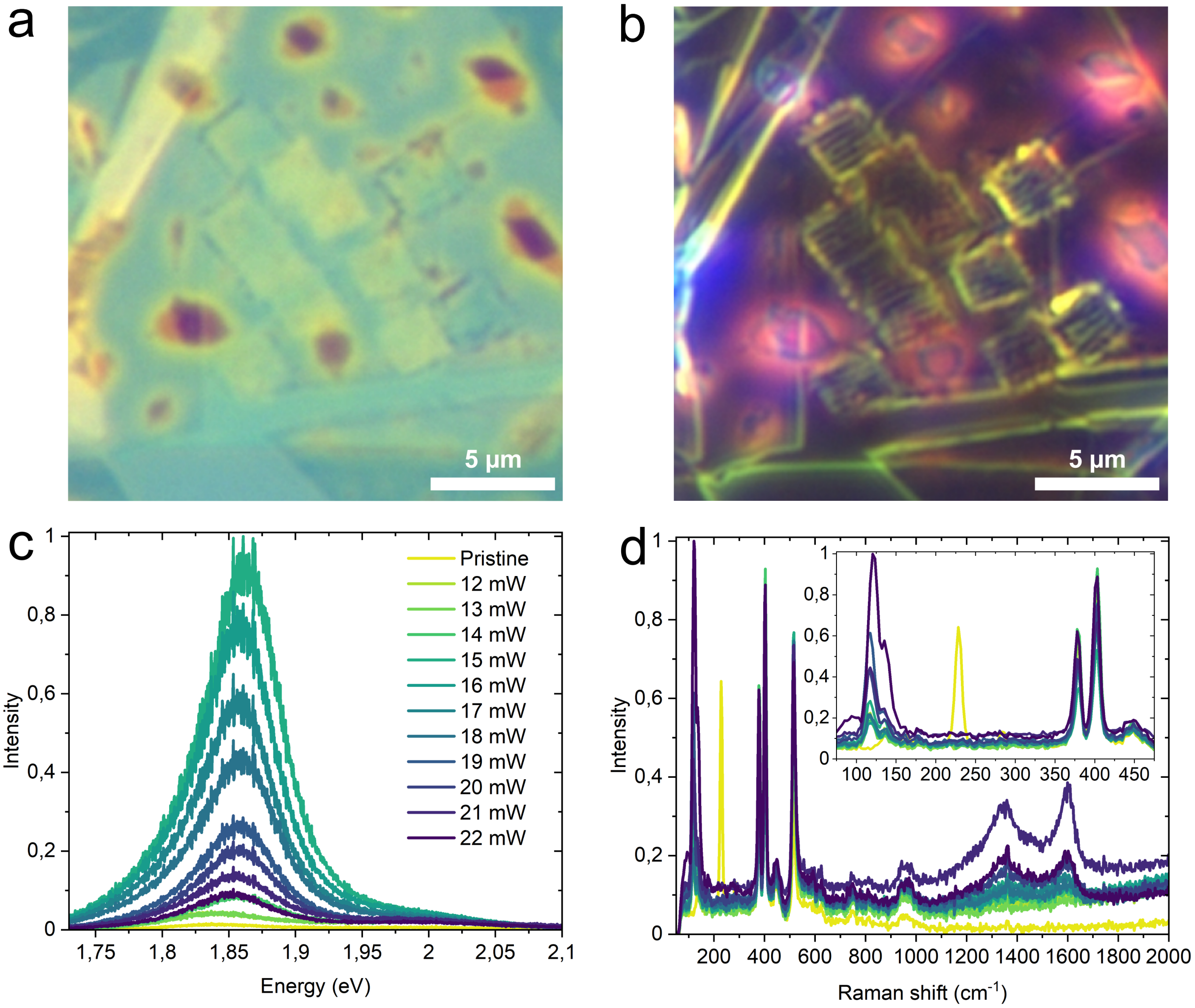

Figure S1: Optical characterization data for the sample used to determine the effect of power in the PL enhancement. **a)** Bright-field optical image of the sample, **b)** Dark-field optical image of the sample, **c)** MoS_2 PL for all the user laser powers, and **d)** Raman spectra for all the used laser powers. Figures **c)** and **d)** share the legend.

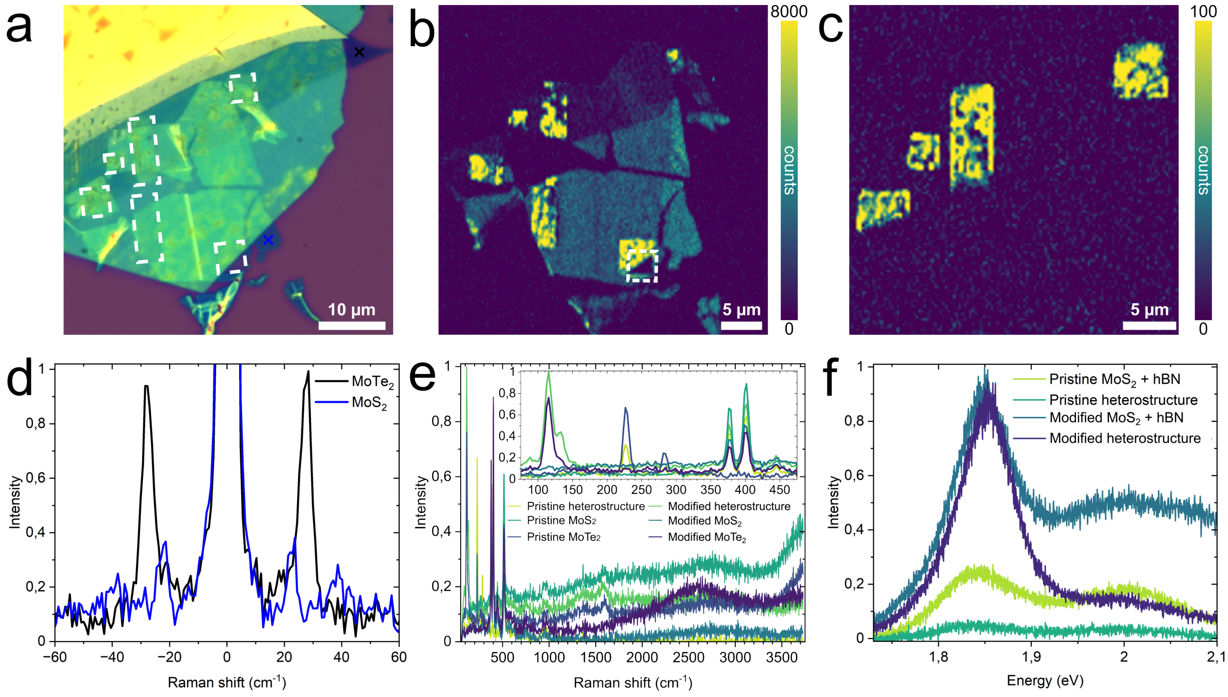

Figure S2: Optical characterization data for the sample used in cross-sectional TEM and EDX measurements. **a)** Optical image of the sample. The white sketched boxes indicate the optically modified areas. **b)** MoS_2 PL map showing multiple modified areas with PL enhancement and PL decrease on the modified MoS_2 area with no hBN cover (indicated by white sketched box), **c)** Te-atom Raman map, **d)** low-frequency Raman spectra of pristine MoS_2 and $MoTe_2$. Based on the spectra both of the flakes are estimated to be bilayer. **d)** Raman spectra of pristine and modified areas in the sample, and **e)** MoS_2 PL spectra of pristine and modified hBN-capped MoS_2 and heterostructure.

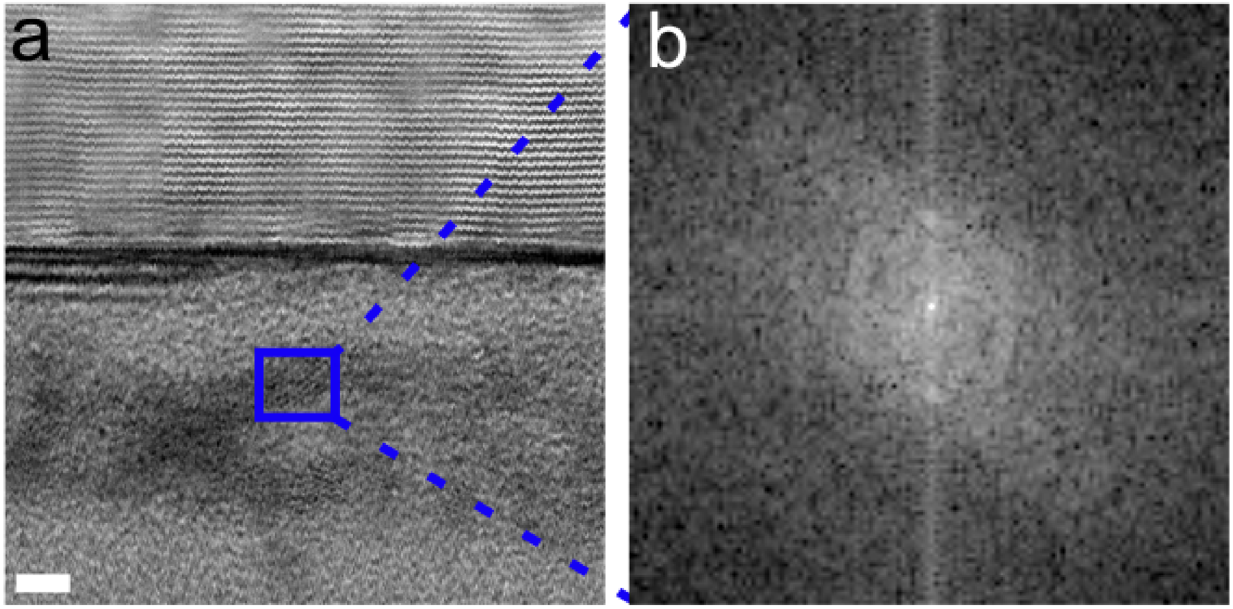

Figure S3: Cross-sectional TEM and FFT results for optically modified $MoTe_2/MoS_2$ heterostructure **a)** TEM micrograph of laser-induced thinning of MoS_2 and deconstruction and clustering of $MoTe_2$. The scalebar is 2 nm. **b)** Fast Fourier transform (FFT) of crystalline material embedded in the SiO_2.

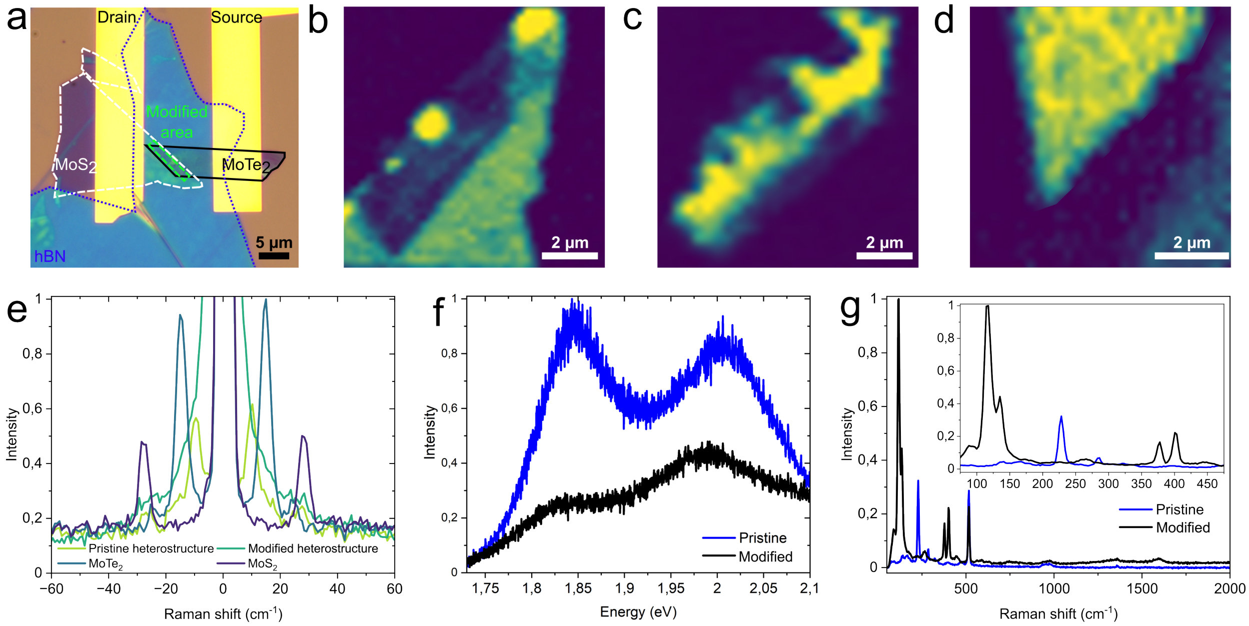

Figure S4: Optical characterization results of the $MoS_2/MoTe_2$ heterojunction used for general electrical characterization. **a)** Optical image of the junction with markings to indicate the flakes, optically modified area, and source and drain electrodes, **b)** MoS_2 PL map of optically modified heterojunction, **c)** Te-atom Raman map of optically modified heterojunction, **d)** Low-frequency Raman map of $MoTe_2$ (peak at $\sim$15.7 cm^{-1}) and MoS_2 (peak at $\sim$28.6 cm^{-1}). Notably there are no discernible peaks at the modified area. **e)** Low-frequency Raman spectra of pristine MoS_2, $MoTe_2$, heterostructure and modified heterostructure. Based on the spectra both of the flakes are estimated to be trilayer, **f)** MoS_2 PL spectra of pristine and modified heterostructure, and **g)** Raman spectra of pristine and modified heterostructure.

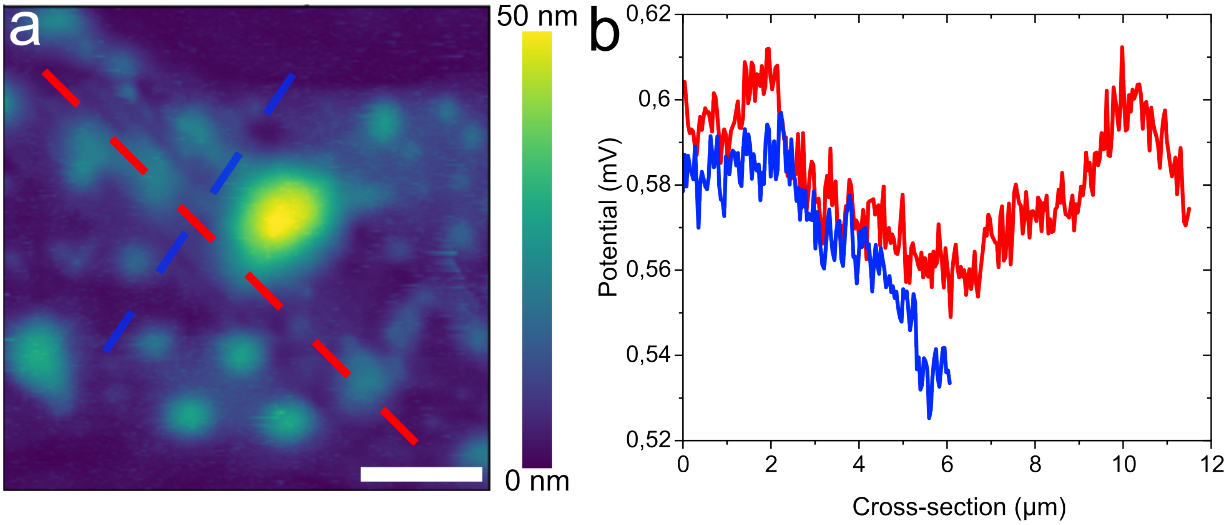

Figure S5: AFM image and KPFM graphs of the $MoTe_2/MoS_2$ heterojunction **a)** AFM height map of the optically modified heterojunction. The scale bar is 5 μm and the dashed lines denote the locations of the KPFM graphs in b), and **b)** KPFM graphs showing potential changes along and across the optically modified heterojunction area.

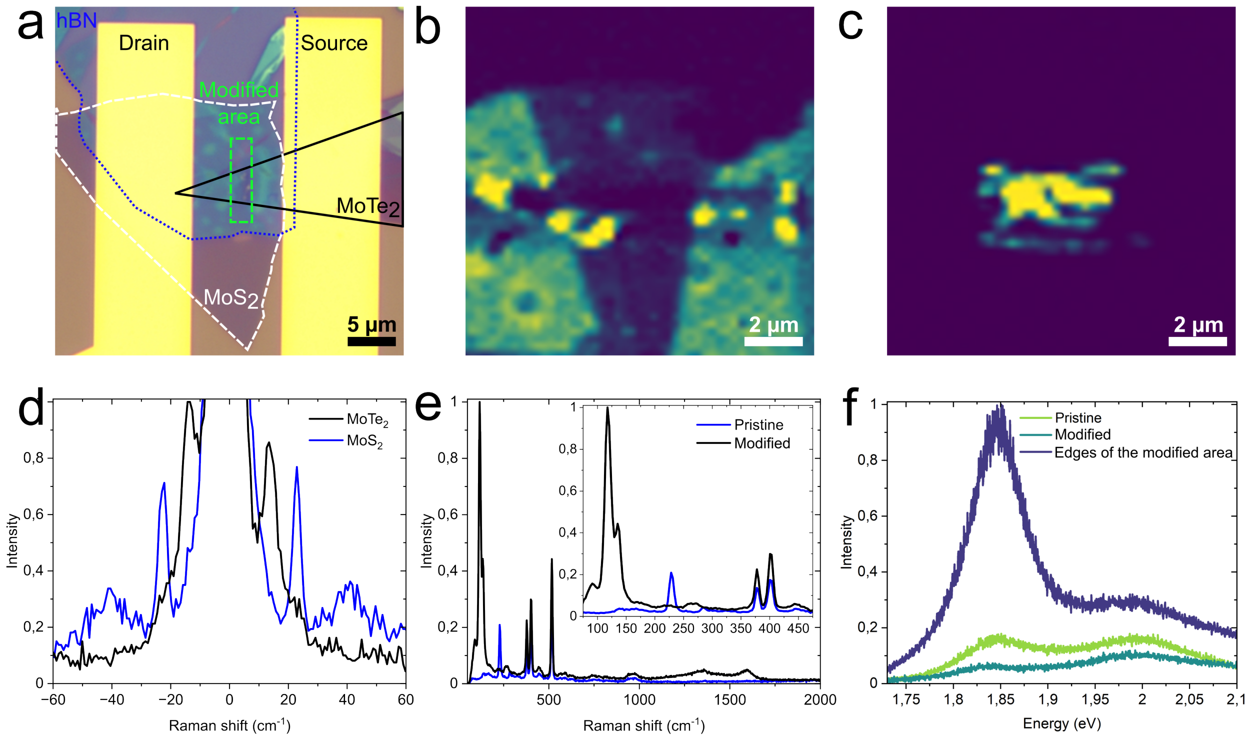

Figure S6: Optical characterization results of the $MoTe_2/MoS_2$ heterojunction used for inverter measurements. **a)** optical image of the junction with markings to indicate the flakes, optically modified area, and source and drain electrodes, **b)** MoS_2 PL map of optically modified heterojunction, **c)** Te-atom Raman map of optically modified heterojunction, **d)** Low-frequency Raman spectra of pristine MoS_2 and $MoTe_2$. Based on the spectra MoS_2 is a bilayer and $MoTe_2$ is a trilayer. **e)** Raman spectra of pristine and modified heterostructure, and **f)** MoS_2 PL spectra of pristine and modified heterostructure.

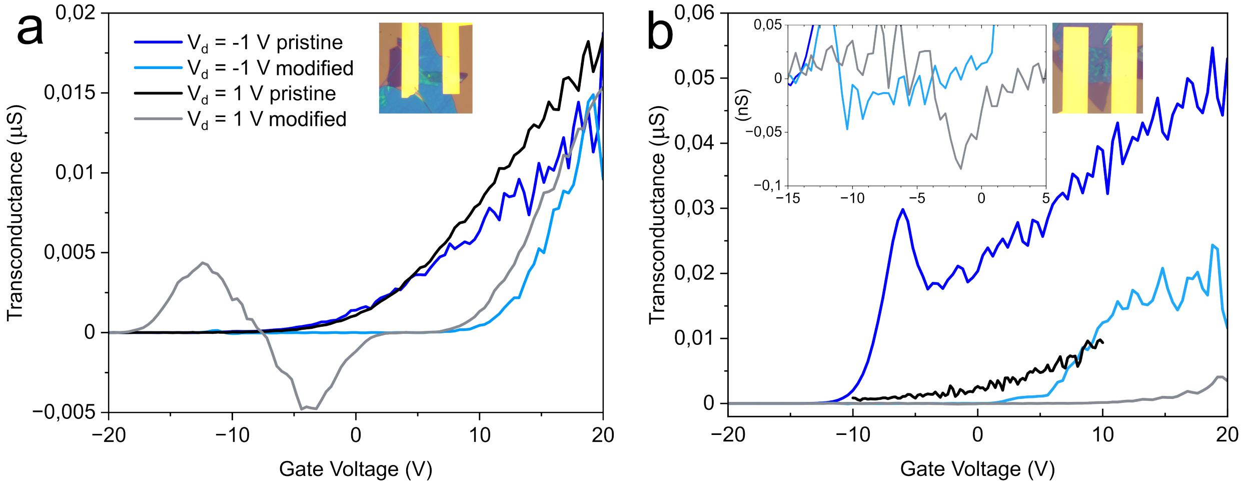

Figure S7: Transconductance plots for both $MoTe_2/MoS_2$ heterojunctions. **a)** Transconductance plots for pristine and modified heterojunction used for electrical characterization, and **b)** Transconductance plots for pristine and modified heterojunction used for inverter measurements. Notably pristine $V_d = 1$ V data was only measured between -10 to 10 V. Both figures share the same legend.

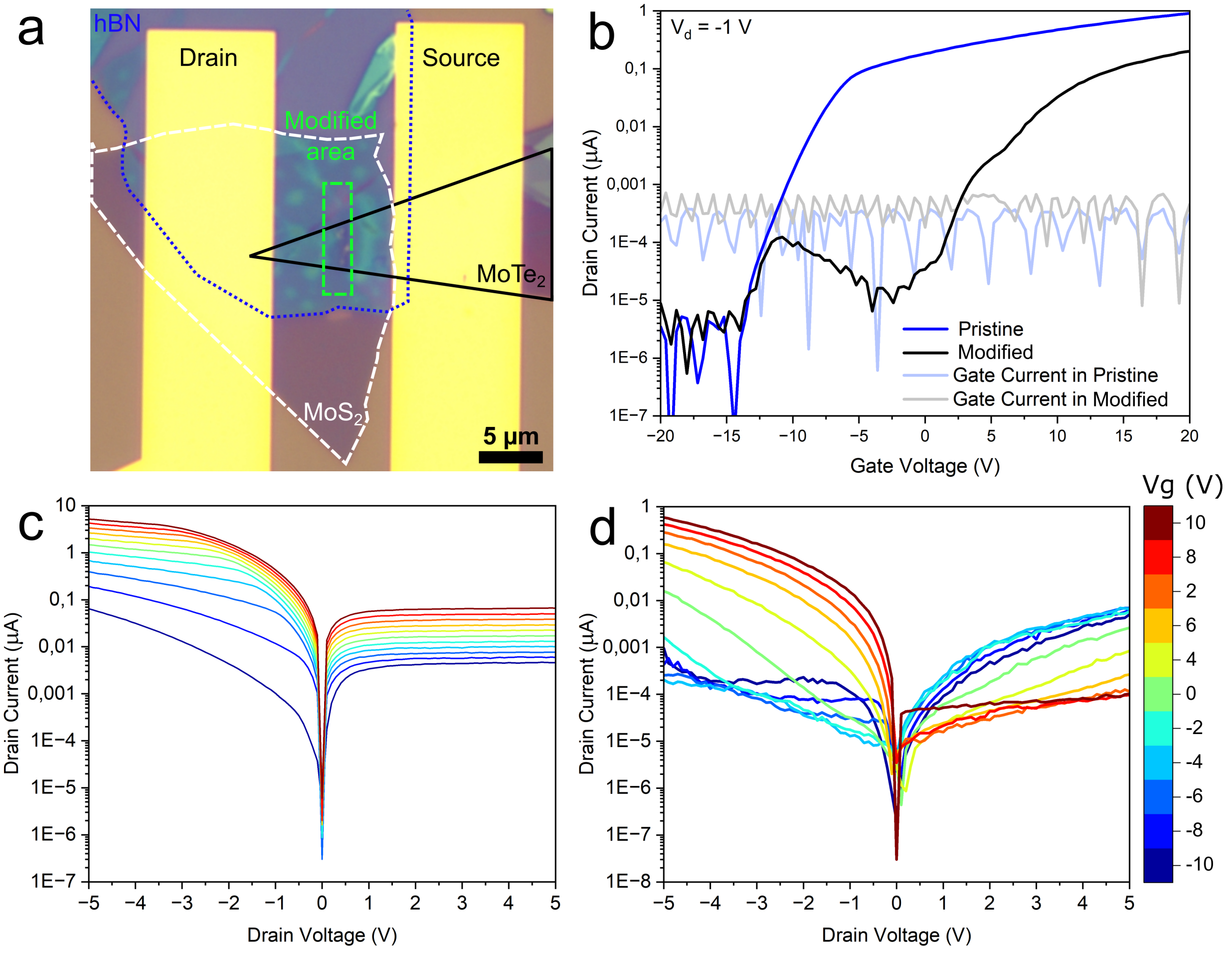

Figure S8: Electrical characterization results of the $MoTe_2/MoS_2$ heterojunction used for inverter measurements. **a)** optical image of the junction with markings to indicate the flakes, optically modified area, and source and drain electrodes, **b)** Transfer curves of the heterojunction before and after optical modification for V_d = -1 V. Gate Currents are included to illustrate that leakage has no effect on the curves. **c)** The output curve of the pristine $MoTe_2/MoS_2$ junction transistor, and **d)** The output curve of the optically modified $MoTe_2/MoS_2$ junction transistor.

The effect of polymer residues on the optical modification

In the article the lack of PL enhancement in the electrical devices was attributed to residues from the electrode lithography process. To prove this, a set of experiments were performed on a singular $MoTe_2/MoS_2$/hBN heterostructure as it was optically modified with 15 mW, 2 s irradiation time and 0.33 μm step size in three different conditions: i) Pristine (after transfer with polydimethylsiloxane (PDMS)), ii) After coating with polymethyl methacrylate (PMMA) photoresist and stripping with acetone and isopropyl alcohol, mimicking lithography process, and iii) After cleaning the sample with oxygen plasma (flow: 50 sccm, RF power: 150 W, time 1 min),

The Raman and PL spectroscopy results, displayed in Figure S9, indicate that the application of photoresist hinders the PL enhancement process, but it can be restored and even improved with oxygen plasma cleaning[6] of the surface. Interestingly, the Raman signal from the Te-cluster is enhanced in the lithography processed samples and the $MoTe_2$ peaks are completely diminished. Additional evidence on the effect of residues can be seen in Figure S9c, which shows the negative optical modification PL results for a heterostructure trans-

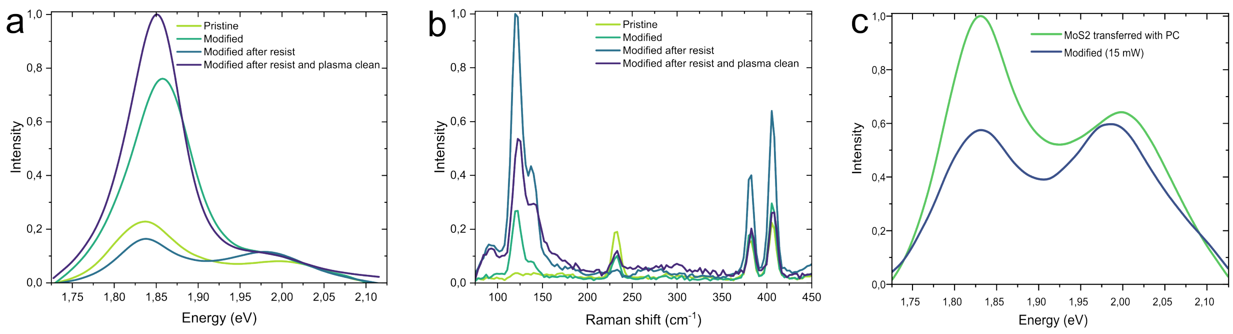

Figure S9: The dependence of optically modified heterostructure's Raman and PL signals on different polymers used during the fabrication process. **a)** Difference in the PL of a $MoTe_2/MoS_2$/hBN heterostructure modified after PDMS-assisted deterministic transfer and after a consequent PMMA resist deposition and stripping, **b)** Difference in the Raman signal of a $MoTe_2/MoS_2$/hBN heterostructure modified after PDMS assisted deterministic transfer and after a consequent PMMA resist deposition and stripping, and **c)** Difference in the PL of pristine and modified $MoTe_2/MoS_2$/hBN heterostructure fabricated by PC assisted deterministic transfer and after a consequent PMMA resist deposition and stripping

ferred with polycarbonate polymer. All this indicates that the conditions on the surface of the sample are important for the optical modification process.

The underlying phenomenon behind this is hard to pinpoint. It is unlikely that the impurities on the top of the sample can affect the TMDs of the heterostructure chemically, although some could diffuse or physisorb to the active materials. The more likely explanation is the amorphous carbon layers typically left from optically burnt polymer residues. Amorphous carbon has been shown to enhance the reflectivity of the surface,[7] which could hinder the access of light to the TMDs or trap light withing, changing the modification dynamics. This is supported by the fact that PMMA and PC, both carbon-based residues, block the PL enhancement, while PDMS, a silicon chain polymer with less organic chains, does not. However, the exact mechanism of the phenomenon requires more detailed investigation and is outside the scope of this research.

The effect of optical modification on hBN

It is also crucial to assess the impact of optical modification on the capping hBN layer to rule out its influence on the observed changes. TEM images of pristine and modified hBN (Figure S10a, b) show no change in height, though surface residues are altered. EDX analysis (Figure S10c) reveals no significant changes in hBN's chemical composition, aside from a slight increase in nitrogen concentration. Similarly, Raman spectra (Figure S10d) show no major variations, aside from the rising amorphous carbon signal. However, residues on modified hBN exhibit diverse chemical compositions, including carbon (likely from polymers), oxygen (from polymers and the environment), copper (from the TEM grid), silicon (possibly sputtered during milling), platinum (from sample coating), and gallium, whose origin remains unclear but may stem from metal evaporation impurities.

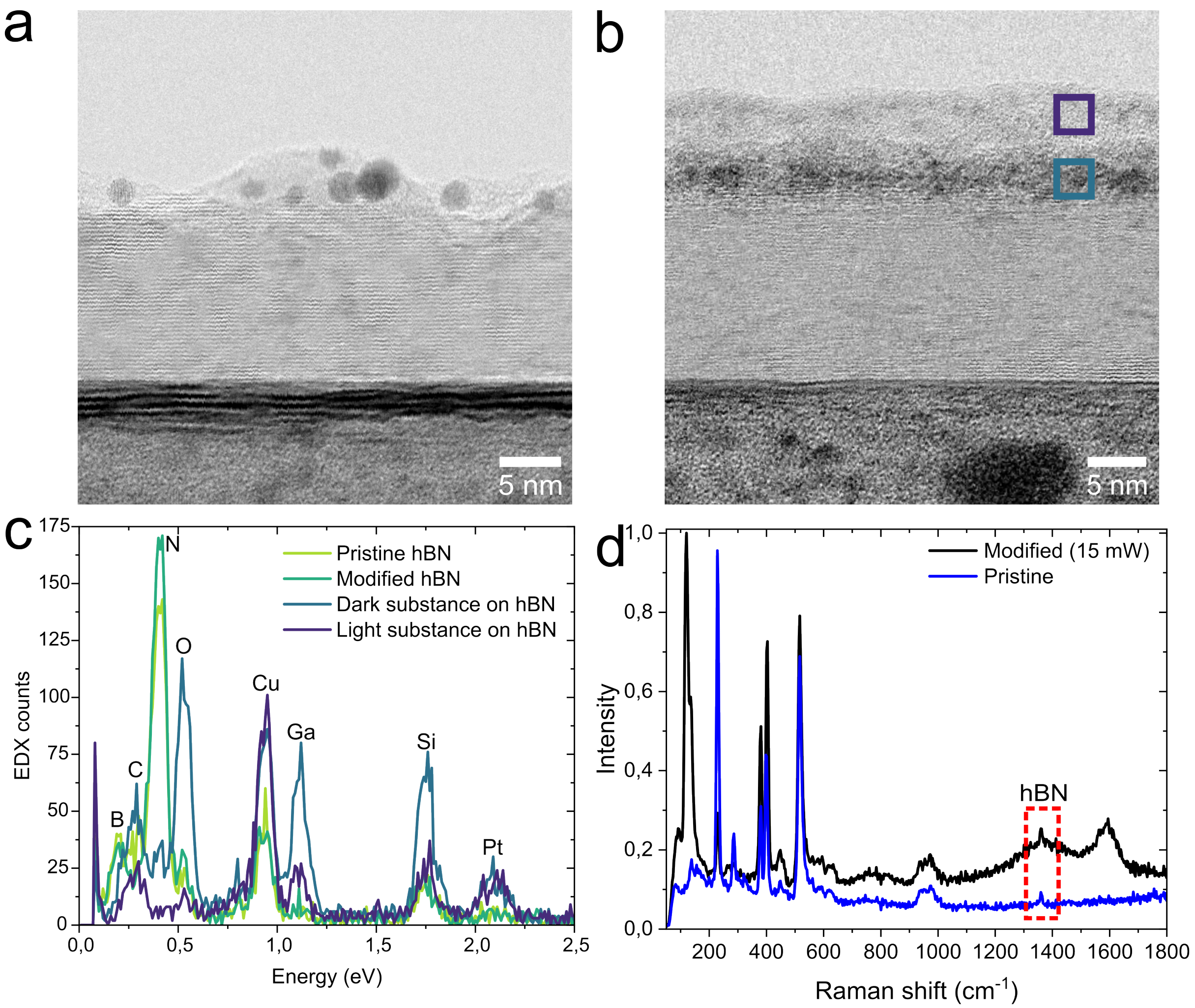

Figure S10: Transmission electron microscopy (TEM), energy dispersive x-ray (EDX) and Raman spectroscopy results of the pristine and optically modified hBN. **a)** TEM image of the pristine heterostructure, **b)** TEM image of the optically modified heterostructure. The colored squares denote the ‘substance’ EDX spectra in c), **c)** EDX analysis of the pristine and modified hBN as well as the dark and light substance on modified hBN, and **d)** Raman spectrum of pristine and optically modified hBN on a heterostructure.

References

(1) Ahmed, F. et al. Deterministic Polymorphic Engineering of $MoTe_2$ for Photonic and Optoelectronic Applications. *Advanced Functional Materials* **2023**, *33*, 2302051.

(2) Kowalczyk, H.; Biscaras, J.; Pistawala, N.; Harnagea, L.; Singh, S.; Shukla, A. Gate and Temperature Driven Phase Transitions in Few-Layer $MoTe_2$. *ACS Nano* **2023**, *17*,

6708–6718.

(3) Kim, E.; Ko, C.; Kim, K.; Chen, Y.; Suh, J.; Ryu, S.-G.; Wu, K.; Meng, X.; Suslu, A.; Tongay, S.; Wu, J.; Grigoropoulos, C. P. Site Selective Doping of Ultrathin Metal Dichalcogenides by Laser-Assisted Reaction. *Advanced Materials* **2016**, *28*, 341–346.

(4) Alrasheed, A.; Gorham, J. M.; Khac, B. C. T.; Alsaffar, F.; Delrio, F. W.; Chung, K. H.; Amer, M. R. Surface Properties of Laser-Treated Molybdenum Disulfide Nanosheets for Optoelectronic Applications. *ACS applied materials interfaces* **2018**, *10*, 18104.

(5) Akkanen, S. T. M.; Arias-Muñoz, J. C.; Emelianov, A. V.; Mentel, K. K.; Tammela, J. V.; Partanen, M.; Das, S.; Faisal, A.; Pettersson, M.; Sun, Z. Enhanced Nonlinear Optical Responses in MoS_2 via Femtosecond Laser-Induced Defect-Engineering. *Advanced Functional Materials* **2024**, 2406942.

(6) Jain, A.; Bharadwaj, P.; Heeg, S.; Parzefall, M.; Taniguchi, T.; Watanabe, K.; Novotny, L. Minimizing residues and strain in 2D materials transferred from PDMS. *Nanotechnology* **2018**, *29*, 265203.

(7) Baydoğan, N. D. Evaluation of optical properties of the amorphous carbon film on fused silica. *Materials Science and Engineering: B* **2004**, *107*, 70–77.
